# SC-VAR: a computational tool for interpreting polygenic disease risks using single-cell epigenomic data

**DOI:** 10.1093/bib/bbaf123

**Published:** 2025-03-24

**Authors:** Gefei Zhao, Binbin Lai

**Affiliations:** Institute of Medical Technology, Peking University Health Science Center, 38 Xueyuan Rd, Hai Dian Qu, Beijing 100191, China; Biomedical Engineering Department, Institute of Advanced Clinical Medicine, Peking University, 5 Yiheyuan Rd, Haidian District, Beijing 100191, China; Institute of Medical Technology, Peking University Health Science Center, 38 Xueyuan Rd, Hai Dian Qu, Beijing 100191, China; Biomedical Engineering Department, Institute of Advanced Clinical Medicine, Peking University, 5 Yiheyuan Rd, Haidian District, Beijing 100191, China; Department of Dermatology and Venerology, Peking University First Hospital, 8 Xishiku Ave, Xicheng Distric, Beijing 100191, China; State Key Laboratory of Molecular Oncology, Peking University International Cancer Institute, 5 Yiheyuan Rd, Haidian District, Beijing 100191, China

**Keywords:** single-cell epigenomics, polygenic disease risks, genome-wide association studies (GWAS), noncoding variant annotation, disease relevance score

## Abstract

Motivation: One major challenge of interpreting variants from genome-wide association studies (GWAS) of complex traits or diseases is how to efficiently annotate noncoding variants. These variants influence gene expression by disrupting *cis*-regulatory elements (CREs), whose spatial and cell-type specificity are not adequately captured by conventional tools like multi-marker analysis of genomic annotation. Current methods either rely on linear proximity to genes or quantitative trait locus (QTL) data yet fail to integrate single-cell epigenomic information for a comprehensive annotation. Results: We present SC-VAR, a novel computational tool designed to enhance the interpretation of disease-associated risks from GWAS using single-cell epigenomic data. SC-VAR leverages single-cell epigenomic data to predict functional outcomes including risk genes, pathways, and cell types for both coding and noncoding disease-associated variants. We demonstrate that SC-VAR outperforms state-of-the-art methods by predicting more validated disease-related genes and pathways for multiple diseases. Additionally, SC-VAR identifies cell types that are susceptible to disease, along with their specific CREs and target genes linked to risk. By capturing a broad range of disease risks across human tissues at distinct developmental stages, SC-VAR could enhance our understanding of disease mechanisms in complex tissues across different life stages.

## Introduction

Genome-wide association studies (GWAS) have identified thousands of variants associated with complex human traits [1–3] However, translating this knowledge into clinical insights or disease-relevant biology remains challenging, primarily because more than 90% of GWAS variants fall in noncoding regions of the genome [4, 5]^.^ These variants might affect gene functions by accumulating in *cis*-regulatory elements (CREs) disrupting binding of transcription factors (TFs) and then affecting gene expression levels [6].

Several methods have been developed to annotate CREs loci, such as GREAT [7] and loci2path [8]. GREAT [7] annotates CREs by analyzing their functional significance related to a gene ontology or a pathway based on the proximity of genes in the genome. The loci2path [8] links loci to genes by incorporating eQTL information. In addition, some models and methods have been developed to convert variant-level risk significance from GWAS to gene- and gene-set-level risk significance, such as multi-marker analysis of genomic annotation (MAGMA) [9], which performs both gene-level association testing and pathway enrichment analysis to uncover potential biological mechanisms underlying complex traits. However, MAGMA employs a window-based strategy to assign variants to nearby genes. Although MAGMA can extend the region to include additional variants, it has obvious limitations in dealing with variants within noncoding regions, owing to the arbitrary setting of the window size: a large genomic window may include many regions without functional connectivity while a small window would miss true distal CREs that regulate genes via chromatin looping but are far away from the gene in the linear distance of genome. Some extensions have been proposed to extend gene variant annotation to provide more powerful functionality using transcriptomics networks (e.g. nMAGMA [10]) or eQTL data (e.g. E-MAGMA [11]). These methods have been widely used to identify novel gene loci and functional pathways that affect complex traits and diseases [12–14].

Besides gene variant annotation approaches based on genomic loci, gene network, and eQTL, epigenomic-based approach is another promising one. It has at least two advantages in explaining the variants in noncoding regions by exploring their regulatory functions. Firstly, epigenomic assays help link distal CREs to target genes reliably and consequently improve the capability to annotate distal disease-related SNPs. For example, one extension of MAGMA based on Hi-C assays (H-MAGAMA) utilizes distal SNPs to explore disease risks by calling SNP-gene links via predicted chromatin loopings of CREs and genes [15]. Secondly, advanced epigenomic techniques such as single-cell genomic assays depict CREs in cell-type-specific manner associated with different tissues and different stages of life time, providing great opportunities to do spatial–temporal interpretation of disease-related SNPs [16, 17] in tissues with high level of cellular heterogeneity, which cannot be achieved by gene network-based or eQTL-based approaches. Recent single-cell epigenomic atlas studies employed methods to assign risk scores to different cell types by estimating the heritability of SNPs residing within cell-type-specific CREs [18–20]. However, the method is still limited to use single-cell epigenomic or single-cell multiome data to annotate noncoding variants in terms of exploring the affected genes and pathways, which is crucial for understanding the molecular mechanisms underlying the genetic basis of polygenetic diseases.

In addition to gene and pathway insights, identifying cell-type-specific contributions is also critical for understanding disease etiology and can provide valuable information for new therapeutic strategies. Methods have been developed to prioritize cell-type risks related to diseases based on single-cell RNA sequencing, such as scRDS [21], which mainly account for variants within gene bodies, but the methods for prioritizing cell-type risks based on noncoding variants are not yet available.

Here we report a human variant interpreter, named SC-VAR, based on single-cell epigenomic data. This tool aims to interpret disease-related risks on three layers: risk genes (individual genes and gene sets), CREs, and cells (individual cells and cell type). SC-VAR extends conventional SNP annotation by linking noncoding GWAS variants to target genes via single-cell ATAC-seq data and enabling the identification of risk SNPs located in cell-type-specific CREs. Unlike previous methods that primarily annotate variants within gene bodies or rely on arbitrary genomic window settings, SC-VAR leverages cell-type and stage-specific SNP-CRE-Gene interactions, offering a refined, biologically informed approach to genome annotation that more accurately reflects the complexity of gene regulation in human disease. When applied with single-cell multi-omics data, SC-VAR further strengthens these linkages by correlating CRE activity with gene expression.

We applied SC-VAR on complex disorders with 12 single-cell epigenomic and multiome datasets from human tissues to examine several complex disorders, including skin diseases, gastrointestinal tract diseases, heart diseases, psychiatric, and neurodegenerative diseases. By correctly assigning more SNPs in CREs, SC-VAR was able to identify more reliable SNP-gene links and detected more risk genes than MAGMA. Several risk genes detected by SC-VAR have been verified to be functionally associated with disease in previous research [22–24]. SC-VAR further highlighted developmental stage-specific risk genes and pathways relevant to psychiatric disorders. By analyzing risk genes and CREs, SC-VAR identifies high-risk cell types within complex tissues which are unexplored by previous GWAS-based methods.

In summary, SC-VAR advances the field by linking single-cell epigenomic data with genetic risk, delivering a robust framework for interpreting the impact of noncoding variants on disease biology across different tissues and developmental stages. This method improves understanding of genetic influences on diseases and provides valuable insights into complex genetic diseases from both gene and cell type perspectives. In summary, we provide a powerful tool to interpret genome-wide risks of SNPs for complex genetic traits or diseases with single-cell epigenomic data.

## Materials and methods

### Genome-wide association studies dataset

The complete set of summary statistics from the GWAS for schizophrenia (SCZ) was downloaded from the fastGWA data portal [25] (https://yanglab.westlake.edu.cn/data.html), which was generated by applying genome-wide association analysis to traits on individuals of European ancestry in the UK Biobank. The dataset contains a total number of 11 842 642 SNPs associated with traits for SCZ. To expand the application scenarios of our method, we additionally used publicly available GWAS summary datasets from FINNGEN R9 version [26]. These datasets include the phenotypes: Attention Deficit Hyperactivity Disorder (ADHD), Bipolar Disorder (BD), Obsessive-Compulsive Disorder (OCD), Alzheimer’s Disease (AD), Parkinson’s Disease (PKS), Autism Spectrum Disorder (ASD), Inflammatory Bowel Disease (IBD), Eczema, and Atrial Fibrillation (AF). Detailed information about these datasets is provided in Supplementary Table S1.

As publicly available GWAS summary statistics were used, no data points were excluded from analysis, and no statistical methods were employed to predetermine the sample size. All data are aligned and annotated or convert to hg38 reference genome.

### Single-cell epigenomic dataset

The scATAC-seq of adult brain data and the multi-omics data of the human neocortex are available in NIH GEO under accession numbers GSE147672 [27] and GSE204684 [28]. The scATAC-seq of fetal brain and heart data can be downloaded from Descartes database (https://descartes.brotmanbaty.org/). The scATAC-seq dataset of human peripheral blood mononuclear cells (PBMCs) is freely available from 10X Genomics (https://www.10xgenomics.com/datasets). The scATAC-seq dataset of human sigmoid colon, small intestine, transverse colon, skin (sun-exposed and normal), and heart data is available under accession number GSE184462 [19].

### Disease risk genes

To validate the accuracy and interpretability of our method for identifying risk genes for SCZ, we collected a list of risk genes from various public resources including CTD, dbGAP, GWASdb, GAD, DisGeNET, PheGenI, GeneRIF, and HuGE. Genes from CTD were selected based on manually curated disease–gene associations from literatures while genes from dbGAP, GWASdb, PheGenI, and GAD were selected based on GWAS or GWAS Catalog SNP-Phenotype associations. The genes selected from GeneRIF and HuGE navigator were obtained based on the genetic association by text-mining. DisGeNET integrates data from expert curated repositories, GWAS catalogues, animal models, and the scientific literature. In total, we obtained a list of 2017 candidate risk genes associated with SCZ after removing duplicates. For IBD, we collected a list of risk genes from IBDDB which is a manually curated and text-mining-enhanced database. The database provides a unique combination of 289 IBD-related genes.

We collected a list of AF risk genes from public resources, similar to those used for SCZ, including CTD, dbGAP, GWASdb, GAD, GeneRIF, and HuGE. After removing duplicates, we compiled a list of 782 candidate risk genes associated with AF after removing duplicates. The same for eczema risk genes from public resources. And we compiled a list of 683 candidate risk genes after removing duplicates (Supplementary Table S2).

### Design of SC-VAR method

**Figure 1:**
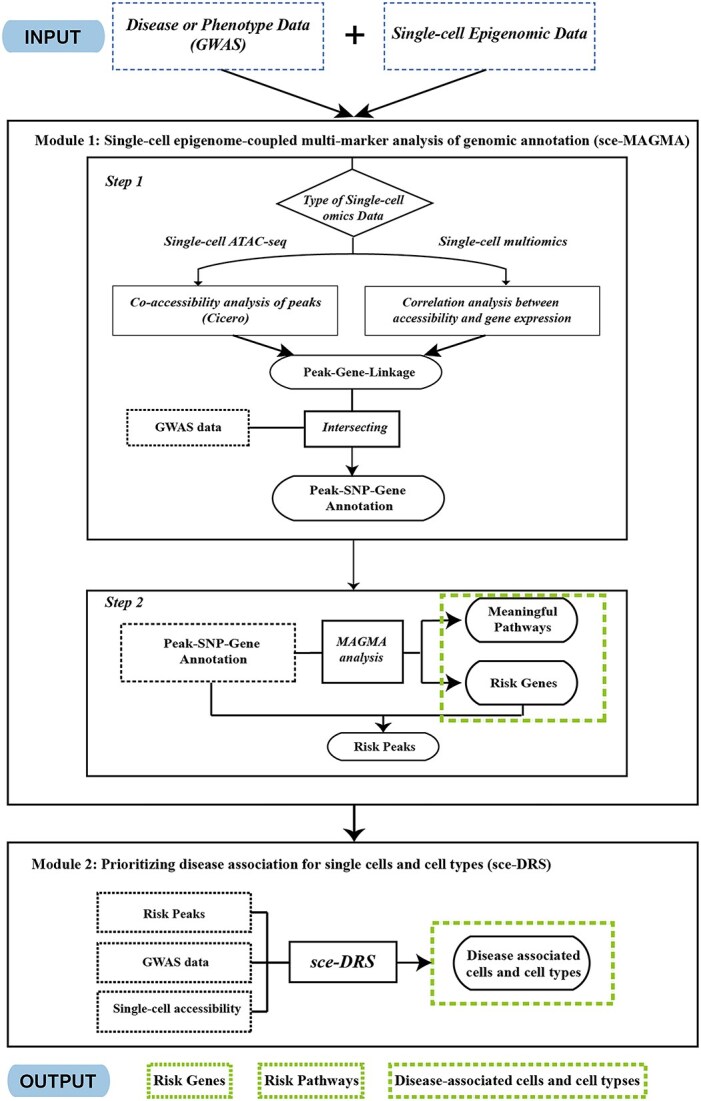
Flowchart of the SC-VAR analytical framework. The process starts with the input of single-cell omics data along with GWAS information related to diseases or phenotypes. Then, SC-VAR employs two modules. The first module sce-MAGMA predicts risk genes, pathways, and CREs. The second module sce-DRS prioritizes disease association for single cells and cell types. The major output of SC-VAR includes risk genes, pathways, and disease-associated cells and cell types.

SC-VAR takes GWAS data and single-cell epigenomic data (scATAC-seq or single-cell multi-omic ATAC + RNA sequencing data) as input and includes two modules to annotate the GWAS variants. The first module is designed to perform single-cell epigenome-coupled multi-marker analysis of genomic annotation (sce-MAGMA) to predict risk genes and gene sets associated with disease or phenotype of interest. This module consists of two steps. The first step establishes the CRE peak-gene connections via the single-cell data and then generates the SNP-gene annotation by aggregating SNPs within the CREs and gene bodies. This step provides a whole genome multi-marker annotation. In the second step, sce-MAGMA applies MAGMA on the SNP-gene annotation to prioritize the disease risks for genes and gene sets. In Module 2, SC-VAR employs an algorithm named sce-DRS (single-cell epigenome-based disease relevance score) to integrate the activity of risk CREs of single cells with the significance of genetic variants from GWAS to prioritize disease-associated cells and cell types within complex tissues at single-cell resolution, independent of annotated cell types. The whole workflow is illustrated in Fig. 1.

#### Module 1. Single-cell epigenome-coupled multi-marker analysis of genomic annotation

Step 1. Linking risk variants to target genes: To determine which SNPs are located within the CREs linked to the genes, we predict the CRE-gene links from single-cell ATAC-seq or multi-omics (single-cell ATAC and RNA sequencing) data. Specifically, for scATAC-seq data we employ cicero [29] method to calculate co-accessibility of the peaks and construct *cis*-regulatory networks. For a given gene, the peaks linking to the peak at the promoter region of the gene within the *cis*-regulatory network are inferred as linked to the gene. For single-cell ATAC and RNA data, we use LinkPeaks function in Signac package, which implements the method based on the correlation between peak accessibility and gene expression described in SHARE-seq [30], to infer peak-gene associations.

We assign two parts of SNPs to genes. The first part of SNPs is those located within the gene body region, which are exactly what traditional MAGMA method include. The second part of SNPs is located within the CREs linked to the genes. Both parts of SNPs are assigned to the gene, and the SNP-gene annotation file is constructed after removing redundant SNPs for downstream analysis. This SNP-gene annotation includes both coding and noncoding variants linked to genes.

Step 2. Running MAGMA gene and gene set analysis: With the SNP-gene annotation containing both coding and noncoding variants, we perform MAGMA for gene and gene-set layers to predict risk genes and risk biological processes or pathways. Briefly, the gene analysis employs a multiple linear principal components regression model on gene-SNP matrix and uses an *F*-test to compute the gene *P*-value. The gene-set analysis tests whether the genes in a gene set are strongly associated with the disease or phenotype of interest within a regression framework. The detailed methods for MAGMA are described previously [9].

#### Module 2. Prioritizing disease association of cells and cell types based on single-cell epigenome-based disease relevance score

To identify cell or cell types relevant to trait or disease of interest, we developed the algorithm, called sce-DRS. The notion and definition of sce-DRS is modified from scDRS [21] which was proposed to measure disease relevance of single cells using single-cell RNA-sequencing data. Briefly, the sce-DRS method tests whether GWAS-weighted accessibility of risk peaks for cells is stronger than other cells, at both individual cell and cell type (a cluster of cells) level.

Specifically, sce-DRS creates a collection of risk peaks those were identified as being associated with risk genes from Peak-SNP-Gene annotation, as output from the first module sce-MAGMA. To maximize power, each risk peak was weighted by its GWAS *Z* score and inversely weighted by its technical noise level. We took the largest SNPs *Z* score under the peaks as the weight. Therefore the putative disease risk peak set was $P=\left\{1,2,\dots, \kern0.5em {n}_{\mathrm{peaks}}\right\}$ with ${Z}_p$as the weight of each peak.

Then, we calculated the disease association score for each cell $c=1,\dots, {n}_{\mathrm{cell}}$ as follows:


$$ \mathrm{Disease}\ \mathrm{score}:{S}_c=\frac{\sum_{p\in P}{Z}_p{\sigma}_{\mathrm{tech},p}^{-1}{X}_{\mathrm{cp}}}{\sum_{p\in P}{Z}_p{\sigma}_{\mathrm{tech},p}^{-1}} $$

where ${X}_{\mathrm{cp}}$ denotes the cell-peak matrix of single-cell ATAC-seq data. The weights vector of the risk peaks is ${\left\{{Z}_p\right\}}_{p\in P}$ and ${Z}_p$ is defined as the largest *Z* score of the SNPs located within the peak $p$. ${\sigma}_{\mathrm{tech},p}^2$ is a vector that represents the technical variance of each CRE across the samples. We estimate the technical variance similarly to previous works [31, 32]. Specifically, we compute the mean and variance for each CRE across all cells in the original non-log-transformed space. Next, we fit a nonlinear tread to the log10-scale variance/mean relationship using local polynomial regression. The estimated trend models the expected technical variance based on the mean accessibility while the observed variance values above this expected trend reflect biological variance. Given this trend, the proportion of technical variance is computed as the ratio of predicted technical variance to observed variance (in the original space). Finally, the technical variance of the log-transformed data ${\sigma}_{\mathrm{tech},p}^2$is computed as the product of the variance of the CRE in log-transformed data and the proportion of technical variance as estimated above.

After obtaining epigenomic-based cell disease scores, sce-DRS tests whether a cell or cell type is significantly associated with disease using the Monte Carlo (MC) test. sce-DRS constructs a control set by randomly selecting peaks matching the mean accessible level and variance of the risk peaks calculated across all cells and let $\pi (p)$be the corresponding matched control peaks. The weights vector of the control risk peak is ${\left\{{Z}_{\pi (p)}\right\}}_{p\in P}$. Let *B* be the number of MC samples of control sets (default 1000). Then for each cell,


$$ {\displaystyle \begin{array}{c}\mathrm{Control}\ \mathrm{score}:{S}_{cb}^{\mathrm{ctrl}}=\frac{\sum_{p\in{P}_b^{\mathrm{ctrl}}}{Z}_{\pi (p)}{\sigma}_{\mathrm{tech},p}^{-1}{X}_{\mathrm{cp}}}{\sum_{p\in{G}_b^{\mathrm{ctrl}}}{Z}_{\pi (p)}{\sigma}_{\mathrm{tech},p}^{-1}},\kern0.5em \forall b\in \left\{1,\dots, B\right\}\end{array}} $$

For each cell $c$ compute cell-level *P*-values using a MC test based on the empirical distribution of the pooled normalized control scores.



$$ {p}_c=\frac{1+{\sum}_{c=1}^{n_{\mathrm{cell}}}{\sum}_{b=1}^B\mathbb{I}\left({S}_c\le{S}_{cb}^{\mathrm{ctrl}}\right)}{1+{n}_{\mathrm{cell}}B} $$

For each cell types, let $t$ be the top 5% quantile of the disease score of cells from the given cell type as the test statistic, and ${t}_1^{\mathrm{ctrl}},\dots, {t}_B^{\mathrm{ctrl}}$be the same test statistics from the control score of the same cells. The MC *P* value for cell type level can be written as:



$$ {\displaystyle \begin{array}{c}{p}^{\mathrm{mc}}=\frac{1+{\sum}_{b=1}^B\mathbb{I}\left(t\le{t}_b^{\mathrm{ctrl}}\right)}{1+B}\end{array}} $$

The main feature of sce-MAGMA is to map noncoding variants to CREs and allocate them to target genes, which efficiently expands the range of the annotated variants than traditional “window-based” strategy. sce-MAGMA predicts CRE-gene links via either co-accessibility among enhancer and promoter peaks from scATAC-seq or the correlation between peak accessibility and gene expression from single-cell multiome data. With more meaningful SNPs assigned to genes, sce-MAGMA is expected to identify more candidate risk genes and gene sets compared to “window-based” strategy. Since some risk peaks and risk genes might be cell-type-specific, another feature of SC-VAR is to identify which cells or cell types are highly associated with the disease. It calculates the weighted accessibility score of risk peaks for individual cells to evaluate their association with the disease. More details about the model and algorithm of SC-VAR can be found in the Materials and methods section. The SC-VAR package has been released on the Pypi website (https://pypi.org/project/sc-var/) and a manual detailing how to use SC-VAR is available on GitHub (https://github.com/gefeiZ/sc_var/).

### Integration of single-cell multiome datasets

Integration of single-cell multiome datasets from six developmental stages was performed using the merge function in Seurat [31] and Signac [33] packages.

### Pseudotime analysis

Pseudotime analysis was performed using scFates [34] package, which combines graph-based algorithms with probabilistic models to assign pseudotime values to each cell. Starting from a root population, the pseudotime analysis revealed distinct branching events corresponding to key developmental milestones.

### Gene ontology analysis

Gene ontology analysis was performed using g:Profiler [35]. Input gene lists were derived from differential risk gene analyses between key cell types, while an adjusted *P*-value threshold (FDR < 0.05) was applied to filter significant terms.

### Estimating type 1 error rate for single-cell epigenome-coupled multi-marker analysis of genomic annotation

We followed the approach described in MAGMA literature [9] to estimate the type 1 error rate for sce-MAGMA and compared with MAGMA. Specifically, random perturbation of SCZ SNPs was performed and sce-MAGMA was applied on perturbated SNPs. Under each perturbation, type 1 error rate was defined as percentage of predicted genes.

## Results

### SC-VAR efficiently assigns SNPs within distal *cis*-regulatory elements to target genes

To evaluate the capability of SC-VAR, we applied it to GWAS data for several different complex diseases (see Materials and methods section), including SCZ, AF, eczema, and IBD. For each disease, we downloaded single-cell ATAC-seq data with profiled single-cell chromatin accessibility landscapes for different disease-related human tissues: brain cerebrum for SCZ, heart for AF, skin for eczema, and digestive system (PBMC, colon, and intestine) for IBD. The data sources are outlined in the Materials and methods section, and a summary of the data is presented in Supplementary Fig. S1A.

**Figure 2:**
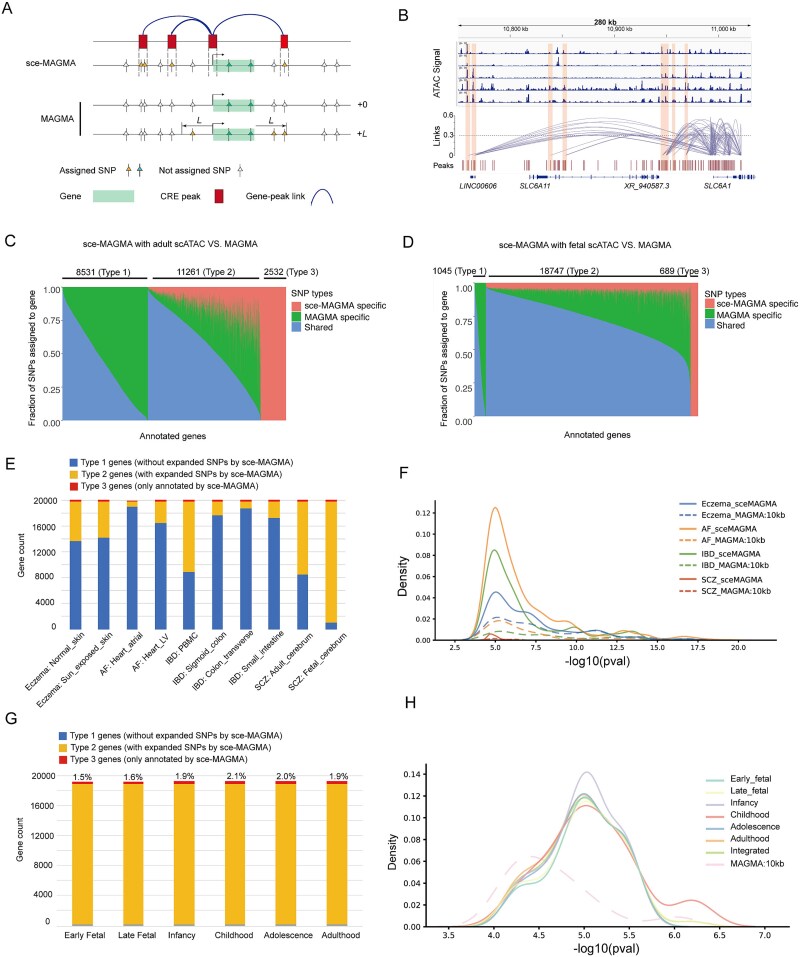
sce-MAGMA efficiently assigns distal noncoding variants to target genes. (A) Schema illustrates strategy of assigning SNPs to target genes for sce-MAGMA and MAGMA. (B) Significant co-accessibility peak links related to gene *SLC6A1*. Accessibility of different cell clusters in scATAC-seq (upper panel) and significant peak links (middle panel) were shown. Distal peaks upstream of gene body region of *SLC6A1* are highlighted. Distribution of different types of SNPs (sce-MAGMA specific, MAGMA specific, and shared SNPs) assigned to genes for analysis with adult (C) and fetal (D) datasets. Three types of genes are defined and indicated. Type 1 genes are those without sce-MAGMA-specific SNPs; type 2 genes are those having sce-MAGMA-specific SNPs and MAGMA SNPs; type 3 genes are those only having sce-MAGMA-specific SNPs. (E) Distribution of three types of annotated genes defined in (C) and (D) derived using multiple human tissue scATAC-seq datasets. (F) The KDE plot illustrates the distribution of SNPs outside gene body annotated to target genes across different diseases. The solid lines indicate annotations made by sce-MAGMA, while the dashed line denotes those by MAGMA. (G) Distribution of three types of annotated genes defined in (C) and (D) derived using multiome ATAC + RNA sequencing datasets from six different developmental stages. (H) The KDE plot illustrates the distribution of SNPs outside gene body annotated to target genes across different development stages.

We first examined SC-VAR’s ability to link SNPs in CREs outside gene body regions to their target genes. The differences between the sce-MAGMA and MAGMA strategies are illustrated in Fig. 2A. In the adult cerebrum dataset for SCZ, for instance, sce-MAGMA assigned 63 (23.5%) SNPs located in the distal peaks to the target gene *SLC6A1* based on co-accessibility between the distal peaks and the peak at the promoter region (Fig. 2B). In comparison, we also used traditional MAGMA method to assign SNPs with two commonly used parameter settings [9]. Traditional MAGMA assigned SNPs using only gene body regions or a 10 kb window around each gene (Fig. 2A). Take the result of SCZ data as an example, we found that sce-MAGMA could annotate additional distal SNPs in a high fraction of annotated genes. Specifically, in the analysis of adult cerebrum data, sce-MAGMA assigned additional distal SNPs for 13 793 genes (61.8%) compared to MAGMA, of which 2532 genes (11.3%) were exclusively annotated by sce-MAGMA (Fig. 2C). In fetal brain cerebrum dataset, sce-MAGMA assigned additional distal SNPs for 19 436 (94.9%) genes, of which 689 (11.3%) genes were exclusively annotated by sce-MAGMA (Fig. 2D). SNPs shared by both methods were generally within gene bodies, while the MAMGA-derived unique SNPs were located within extended 10 kb region upstream of or downstream from the gene body, but these SNPs were not located within the CRE peaks linked to genes. (Fig. 2A).

We further tested SC-VAR on other studied diseases. Results showed that SC-VAR significantly expanded the range of assigned SNPs for a large proportion of genes across most datasets (type 2 and type 3 genes in Fig. 2E). We also examined the significance of these SNPs, especially for those in distal CREs. The results showed that sce-MAGMA annotations for distal SNPs had a consistently higher density in regions with larger logarithm *P*-values for all datasets compared to MAGMA 10 kb extension annotations, suggesting that our method captured more distal SNPs with significant association with diseases.

Next, we assessed an alternative SC-VAR strategy that leverages correlation between CRE accessibility and gene expression, specifically for single-cell multiome ATAC and RNA data (see Materials and methods section). We downloaded six single-cell multiome data of human neocortex from different developmental stages [28] and applied sce-MAGMA analysis to SCZ-related GWAS data. Results showed that sce-MAGMA consistently assigned additional SNPs in distal CREs for the majority of genes (type 2 and type 3 genes in Fig. 2G), uniquely annotating 1.5%–2.1% of genes (type 3 genes) in each dataset. Moreover, examining the significance of these sce-MAGMA-specific distal SNPs across developmental stages revealed similar patterns to those in other tissues (Fig. 2H). These findings suggest that SC-VAR efficiently annotates more significant SNPs distal from genes, resulting in a greater number of candidate genes for downstream analysis compared to MAGMA.

### SC-VAR improved the ability to identify stage-specific or site-specific risk genes through *cis*-regulatory elements information

We then applied SC-VAR to predict disease-related genes using scATAC-seq data from specific tissues and compared the results with MAGMA. Although we cannot directly determine the true disease-related genes, we curated a list of validated genes from external public database that are either directly or indirectly associated with the phenotypes of interest for evaluation (Supplementary Table S2, see Materials and methods section).

**Figure 3:**
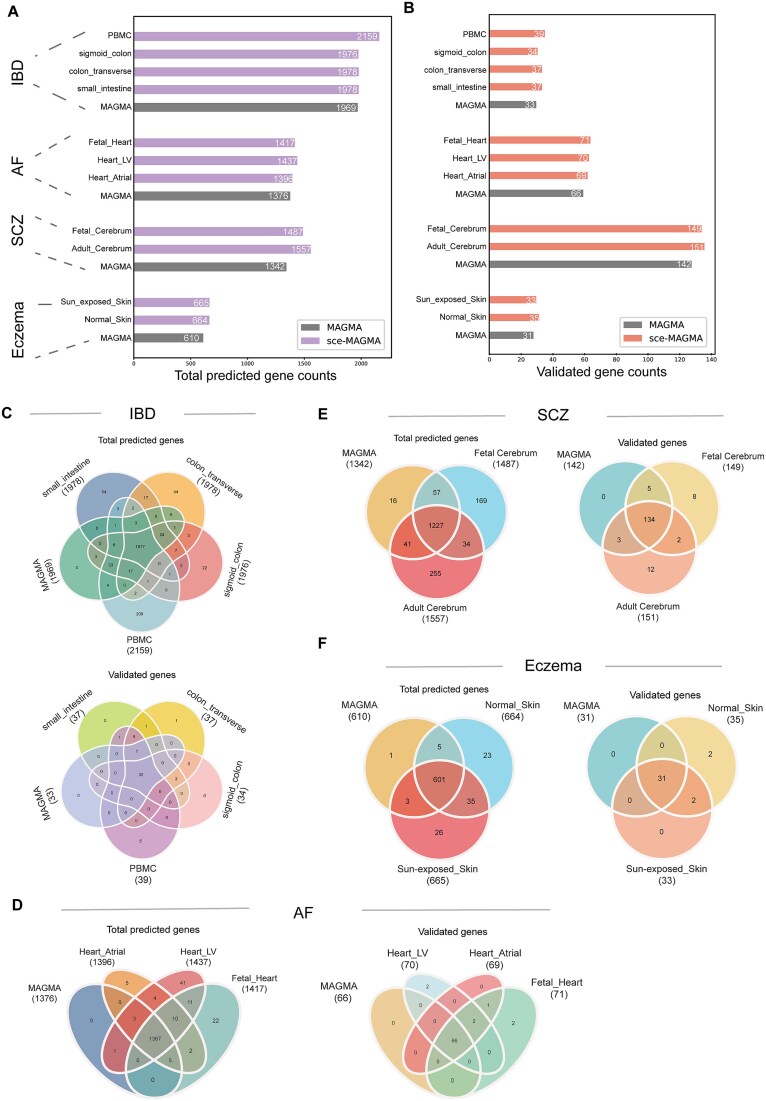
SC-VAR improved the ability to identify stage-specific disease-risk genes. (A) and (B) Bar plot of total predicted genes (A) and validated genes for different diseases (B). (C) Venn diagrams of predicted genes (top) and validated genes (bottom) for IBD. (D)–(F) Venn diagrams of predicted genes (left) and validated genes (right) for AF, SCZ, and eczema.

As shown in Fig. 3A and B, sce-MAGMA identified more significant genes and verified genes across all disease contexts than the classical MAGMA approach, highlighting its enhanced sensitivity in detecting relevant genes for complex diseases. Further, the Venn-diagrams of predicted genes by different approaches revealed that sce-MAGMA almost covered all the predicted genes by MAGMA for different datasets (Fig. 3C–F). This improvement is largely due to sce-MAGMA’s ability to include SNPs in noncoding CRE regions, which are not considered by classical MAGMA.

**Figure 4:**
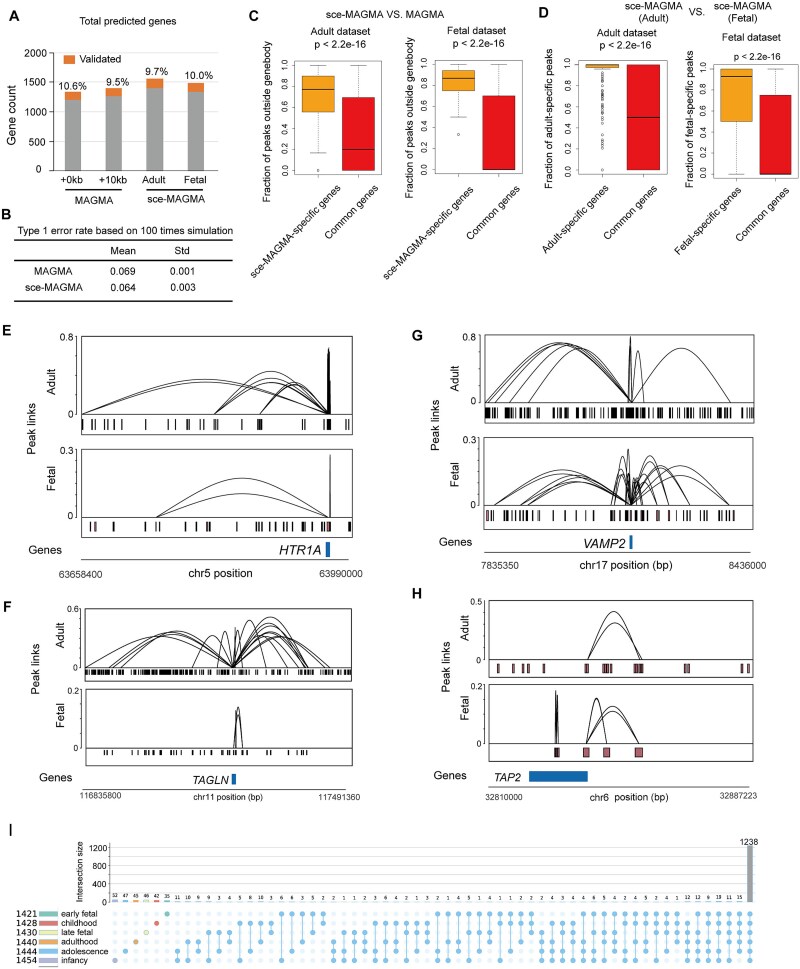
Comparison of gene prediction and validation across different annotation methods. (A) Counts of predicted genes for four approaches, validated genes are highlighted, and fractions of validated genes are labeled. (B) Type 1 error rate comparison between sce-MAGMA and MAGMA. (C) Proportion of CRE-linked peaks outside gene bodies in sce-MAGMA stage-specific genes versus in common genes shared between sce-MAGMA and MAGMA. *P* values were calculated using K–S test. (D) Higher fractions of stage-specific peaks linked to stage-specific genes. *P* values were calculated using K–S test. (E)–(H) Examples of adult-specific gene *HTR1A* (E) and *TAGLN* (F) with stage-specific peak links. Fetal specific gene *VAMP2* (G) and *TAP2* (H) with fetal stage-specific peak links. (I) Upset plot of unique and shared risk genes at six developmental stages for cortex multiome data.

Since MAGMA can extend the “window size” to include SNPs beyond the gene body and account for noncoding variants, we compared sce-MAGMA with MAGMA using a 10 kb extension around the gene body [9–11] on SCZ GWAS data. Our results showed that sce-MAGMA, for both adult and fetal datasets, predicted more significant disease-risk genes and validated genes compared to MAGMA with a 10 kb extension did (Fig. 4A). Next, we estimated the type 1 error rate by randomly perturbating the positions of the SNPs related to SCZ. Results show that sce-MAGMA had similar mean type 1 error rate (0.064) as MAGMA did (0.069) (Fig. 4B).

It is of note that the prediction of sce-MAGMA yielded tissue-specific disease-risk genes, which were highly dependent on varied activity of the CREs in different tissues (Fig. 3C–F). For example, in the result of SCZ datasets, genes uniquely predicted by sce-MAGMA had a higher fraction of linked CRE peaks outside gene body regions compared to genes shared with MAGMA predictions (Fig. 4C). This suggests that these sce-MAGMA-specific genes are mainly influenced by SNPs in distal CREs. When comparing adult and fetal datasets, we found that 19.0% of predicted genes and 10.0% of validated genes were specific to the adult stage, while 15.2% of predicted genes and 9.4% of validated genes were specific to the fetal stage (Fig. 3E). Moreover, adult-stage-specific genes have higher fraction of adult-specific peaks linked to the genes than common genes in adult stage, and fetal-specific genes have higher fraction of fetal-specific peaks linked to the genes than common genes in fetal stage (Fig. 4D), suggesting the risk of stage-specific genes is mainly contributed from SNPs located in stage-specific CREs. For example, the gene *HTR1A* and *TAGLN* are adult-specific validated genes that have adult-specific peak links (Fig. 4E and F) while the gene *VAMP2* and *TAP2* are fetal-specific validated genes that have fetal-specific peak links (Fig. 4G and H). The 5-HT dysfunction has been demonstrated to contribute to not only emotional difficulty but also cognition deficit [36]. *HTR1A* is the most abundantly expressed 5-HT receptor subtype in the mammalian brain and have been found to play important roles in the development and treatment of SCZ [37, 38]. The *VAMP2* gene also known as Synaptobrevin-2 is primarily involved in the fusion of neuronal synaptic vesicles with the cell membrane, and its aberrant function can disrupt human neural development [39–41]. *VAMP2* has also been found to be involved in the regulation of synaptic plasticity [42, 43], suggesting that it may play an important role in the development of SCZ. Our analysis provides the clues implying that the variants on the distal CREs might influence the genes in SCZ in a developmental-stage-dependent manner.

Furthermore, applying SC-VAR to single-cell multi-omics data from the human neocortex at different developmental stages detected SCZ-risk genes that covered most of the genes derived from scATAC-seq data of the adult and fetal samples. As expected, we observed some stage-specific risk genes. Meanwhile, 1238 (69.7%) of risk genes are common across stages (Fig. 4I), such as *FOXP2*, *ERBB4*, and *NRXN1*, consistently implicated in SCZ susceptibility across all stages.

In conclusion, SC-VAR enhances the identification of risk genes by incorporating CREs specific to developmental stages, demonstrating its superiority over the traditional MAGMA approach.

### SC-VAR identifies biological processes or pathways related to disease risks

Since the risk of an individual for a particular GWAS disease or trait is the output of combinatorial effects of multiple SNPs and genes, we extended the evaluation of risk of single genes to meta-genes or gene sets. SC-VAR follows the similar algorithm as MAGMA to calculate the significance of a gene set for disease association, differing only in the gene-SNP annotation (Materials and methods section). Based on the results of detailed gene analysis above, we further explored the gene-sets enriched in those genes to see how the genes are involved in SCZ. We tested the association of gene sets from the MSig7.5 database with the SCZ by both SC-VAR and MAGMA. Many gene sets are detected by both methods as having significant connection with SCZ. However, while MAGMA exclusively detected some gene sets with general biological functions or pathways such as protein import, metabolic process, and receptor signaling, SC-VAR uniquely identified pathways more specifically linked to SCZ (Table 1 and Supplementary Table S3). For example, the response to amyloid beta process is detected as a risk pathway by SC-VAR at the adult stage. In studies of SCZ, amyloids are indicators of cognitive impairment [44]. Notably, SC-VAR also pinpointed the process of snoRNAs as the risk gene set in fetal sample. Previous studies have reported the change of the snoRNA level in schizophrenic patients, which is thought to be related to the dysregulation of alternative splicing in SCZ [45]. Another biological process of note detected by SC-VAR is the regulation of synaptic vesicle exocytosis. Synaptic proteins regulate a number of vesicles that can undergo cytokinesis, and it has been reported that there is a significant reduction in synaptic proteins in patients with SCZ [46–48]. Our data support that neurotransmitter transmission failure at the synapse may lead to SCZ. These results suggest that SC-VAR can uncover putative pathways and biological processes potentially implicated in variant-driven diseases.

**Table 1:** Selected risk biological processes detected by sce-MAGMA

Stage	GOBP	No. of genes	*P*-value	Reference^a^
Adult	Regulation of synaptic vesicle exocytosis	50	6.8E-4	Egbujo *et al*. [46]
	Regulation of DNA metabolic process	378	1.9E-3	Parellada *et al*. [73]
	Regulation of anoikis	24	3.4E-3	Jarskog *et al*. [74]
	Response to amyloid beta	51	4.3E-3	Albertini *et al*. [44]
Fetal	Regulation of metal ion transport	365	1.4E-3	Schoonover *et al*. [75]; Mealer *et al*. [76]
	Macromolecule catabolic process	1226	1.7E-3	Trubetskoy *et al*. [2]
	sno(s)RNA metabolic process	14	1.8E-3	Gibbons *et al*. [45]
	RNA phosphodiester bond hydrolysis	136	2.0E-3	Ermakov *et al*. [77]; Parshukova *et al*. [78]
	Regulation of synaptic vesicle exocytosis	49	2.5E-3	Egbujo *et al*. [46]; Osimo *et al*. [47]; Radhakrishnan *et al*. [48]

^a^References with evidence related to SCZ.

### Linking high-risk cell types from diverse tissues to disease

**Figure 5:**
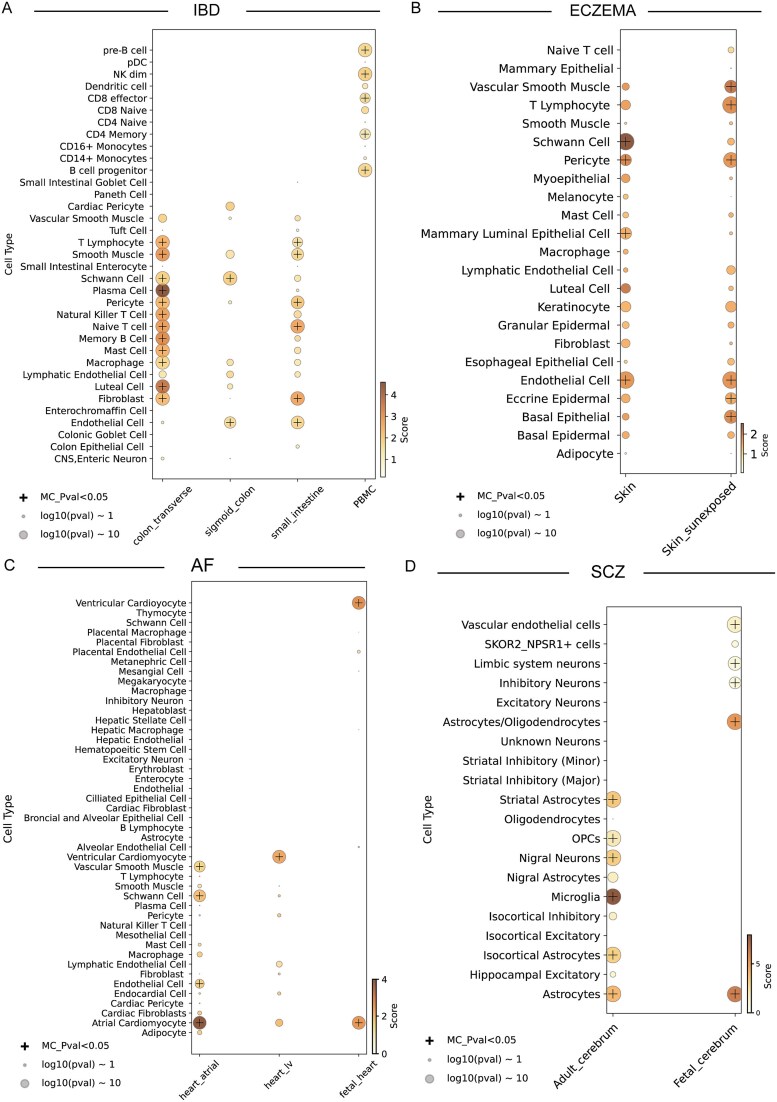
Cell type-specific associations for disease-associated variants across tissues and developmental stages. Bubble plots showing disease association scores of cell types across different tissues and developmental stages for four complex diseases: (A) IBD, (B) eczema, (C) AF, and (D) SCZ. Each panel represents one disease, displaying specific cell types along the y-axis and tissue or body part on the x-axis. Only cell types with MC *P*-values less than .05 are labeled “+” on the plots.

Within a complex tissue consisting of distinct cell types, the impacts from disease-relevant variants are different across cell types and cell states due to their specific transcriptomic and epigenomic profiles. The module sce-DRS of SC-VAR is specifically designed to prioritize disease risks at single-cell and cell-type level (see Materials and methods section). Using GWAS data from IBD, eczema, SCZ, and AF, we evaluated the association of various cell types with these diseases (Fig. 5A–D).

For IBD, significant enrichment was observed in multiple cell types across gut regions (e.g. transverse colon, sigmoid colon, small intestine) and PBMC (Fig. 5A). Plasma cells, smooth muscle cells, and T lymphocytes showed the strongest associations, reflecting the autoimmune nature of IBD [49]. T lymphocytes and plasma cells are considered key players in the intestinal immune system and their dysregulation, leading to excessive immune responses, is a major contributor to the chronic inflammation observed in IBD [50]^.^ In eczema, skin cell types such as basal epidermal, eccrine sweat epidermal, and granular epidermal cells, along with T lymphocytes, were notably enriched (Fig. 5B). Eccrine sweat epidermal and T lymphocytes were specifically enriched in sun-exposed skin, which is interesting. Sweat secretion, as an integral part of the skin’s protective barrier, plays a crucial role in maintaining barrier homeostasis [51]^.^ Previous studies have suggested that sweat management could serve as a potential therapeutic approach for skin dermatitis [52]^.^ Our findings reveal the specific role of eccrine sweat epidermal cells in sun-exposed skin, demonstrating the advantage of using tissue-specific data to uncover novel insights into disease pathophysiology.

For AF, significant associations were identified in heart-related cell types (Fig. 5C). The strong link between ventricular cardiomyocytes and AF is consistent with the electrical dysregulation characteristic of the condition [53]^.^ And the identification of AF-associated cell types aligns with findings from chromatin accessibility profiles, which demonstrated strong enrichment of AF-associated SNPs in cardiomyocyte-specific open chromatin regions [19, 54, 55]. Both our results and these studies highlight the critical role of noncoding SNPs in mediating disease risk. In SCZ, neuronal cell types from both fetal and adult cerebrum were significantly enriched in high risks (Fig. 5D). Astrocytes and microglia were also implicated, underscoring the importance of glial and neuronal interactions in the brain’s pathology [56]. Notably, SCZ displayed substantial within-cell-type heterogeneity, particularly among neuronal and glial cells, suggesting a diverse set of mechanisms involved in the disease’s development.

### SC-VAR reveals dynamic genetic susceptibility across high-risk cell types for brain disorders during brain development

**Figure 6:**
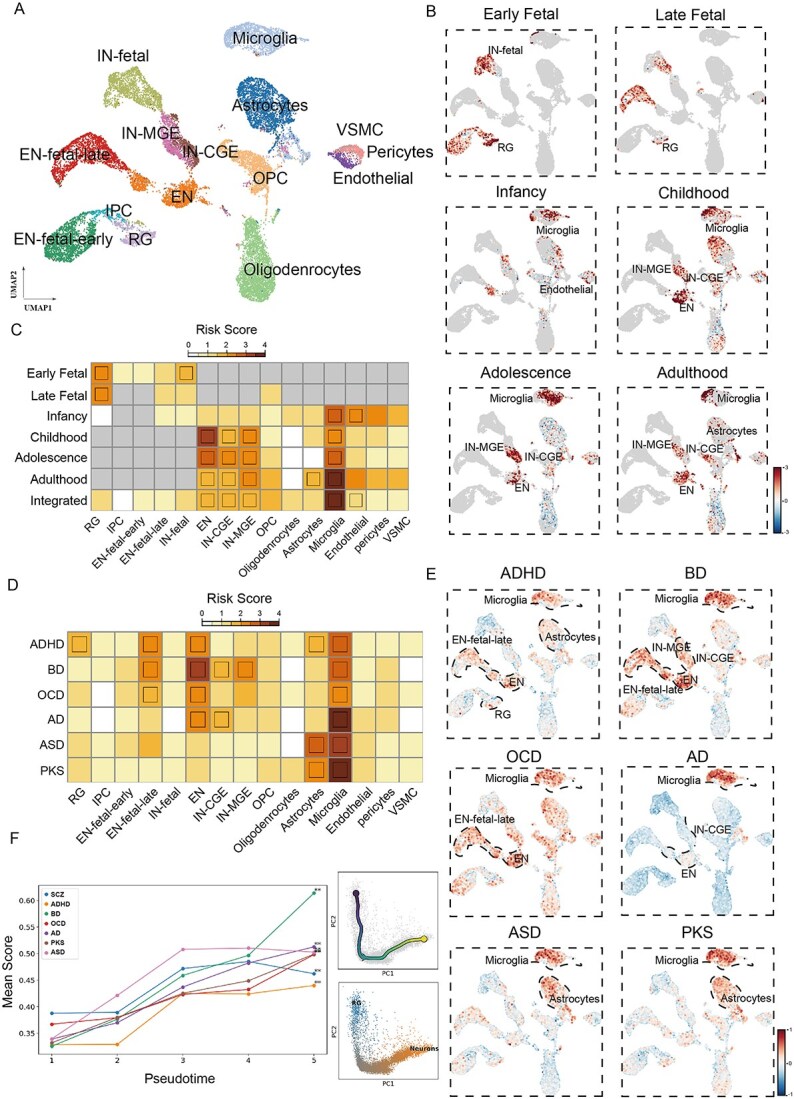
Various disease risk scores of single cells for neocortex samples. (A) UMAP of multi-omics data, showing distinct groups labeled by cell types. (B) Risk scores of single cells at individual developmental stages, significantly associated cell types are labelled on the graph. (C) Heat map shows statistical significance of cell-type associations with SCZ for individual brain development stages and integrated landscape. (D) Heat map shows statistical significance of cell-type associations with different brain disorders for integrated landscape. (E) Risk scores of single cells at integrated landscape of neocortex samples for six different brain disorders. The boundaries of the significant associated cell types are labeled in dash lines. (F) Line plot (left panel) shows increased association score for different brain disorders along pseudo-time trajectory indicating development from RG to mature neurons (right panel). The x-axis denotes time quintile bins, and the y-axis denotes the average sce-DRS disease score in each bin for each disease. *P < .05 and, **P < .005 (one-sided MC test).

Next, we conducted a detailed analysis of finely divided multi-omics brain single-cell data at different developmental stages. We applied sce-DRS on single-cell multiome ATAC + gene expression datasets for neocortex samples derived from different developmental stages (Fig. 6A). By integrating data across developmental stages, we observed dynamic changes in disease risk relevance among neocortical cell types (Fig. 6B and C). In early and late fetal stages, radial glia (RG), as neural progenitor cells, exhibited the highest risk relevance, reflecting their critical role in early brain development and susceptibility to disruptions during this period [57]^.^ As the neocortex matured, microglia displayed the highest risk scores across all cell types, highlighting their involvement in maintaining neuroimmune homeostasis and synaptic pruning [58, 59]. Mature neuronal cell types, including excitatory (EN) and inhibitory neurons (IN), also showed significantly higher risk levels, particularly after the childhood stage, underscoring the importance of excitatory/inhibitory (E/I) balance and neuronal connectivity in SCZ pathogenesis [60, 61].

We then aligned risk cell types with cell-type-specific risk genes and CRE peaks to elucidate the genes and regulatory foundations relevant to high-risk cell types. Our analysis focused on four major high-risk cell types: excitatory neurons (EN), IN-MGE, IN-CGE, and microglia (Supplementary Fig. S1A). EN-specific risk genes (e.g. *RYR2*, *CCKBR*, and *CACNA1C*) are enriched in the Ras-mitogen activated protein kinase (MAPK/ERK) pathway, which facilitates Ca^2+^ flux through NMDA receptors and is crucial for the expression of numerous key downstream genes in neurons [62–64]. This result is in line with the report that NMDA receptor dysfunction plays a significant role in the pathogenesis of SCZ [65, 66]. IN-MGE-specific risk genes (e.g. *FGF14*, *GABRA5*, and *ZNF2)* and IN-CGE-specific risk genes (e.g. *GRIK3*, *PTPN5*, and *BSN*) are enriched in pathways related to GABAergic synapse. Microglia-specific risk genes (e.g. *RGS10*, *TNFRSF1A*, and *MYCBP2*) were enriched in pathways associated with neurotrophic factors, such as Fcγ receptors, which play important roles in the generation, maturation, and integration of new neurons into neural circuits during neurogenesis [67–69]. These results highlight the pathways contributing to cell-type-specific risk in SCZ.

As a proof of principle and to assess the robustness of SC-VAR in elucidating cell types involved in the pathogenic mechanisms of brain diseases, we applied SC-VAR to GWAS data from six brain disorders in addition to SCZ: ADHD, BD, AD, ASD, OCD, and PKS, to evaluate cell-type-specific risk. Our analysis identified 20 significantly associated disease-cell type pairs (Fig. 6D and E), including known associations such as high risks of multiple neuronal subtypes for BD and ADHD [20], and high risks of glial cells for AD and PKS [70, 71]. Additionally, we uncovered several associations that were less clear in existing genetic data but are biologically plausible, like glial cells for compulsive disorder (OCD) and ASD, and neurons for AD.

The association of neurons in AD is consistent with recent studies by Mathys *et al*. [72] using single-cell transcriptomics datasets from Alzheimer’s patients and controls. In these studies, neurons, which are early targets of tau pathology and amyloid-beta accumulation, experience degeneration leading to synaptic dysfunction and compromised neural circuit integrity. Disrupted inhibitory signaling further destabilizes neural circuits, potentially accelerating disease progression. Notably, microglia show high risk score in all studied brain disorders, highlighting the critical roles of neuroinflammation and glial dysfunction in brain disease pathology.

We also traced the developmental trajectory of neuron cells from RG to all mature neurons using pseudotime analysis, exploring the dynamics of risk scores for each brain disorder along the developmental pseudotime (Fig. 6F). The results revealed strong associations with all these disorders (*P* < .005, MC test), indicating a consistent pattern of increased risk scores along the pseudotime and suggesting that mature neurons are more susceptible to polygenic factors.

## Discussion

In this study, we introduce SC-VAR, a software designed to enhance our understanding of the genetic basis of complex diseases. SC-VAR integrates single-cell data to offer a comprehensive genomic analysis that encompasses not only coding regions but also the extensive noncoding regions of the genome. Through extensive evaluation on multiple diseases, we demonstrate that SC-VAR is well-calibrated and robust under realistic scenarios. Leveraging single-cell and multi-omics sequencing technologies, SC-VAR effectively identifies disease-associated genes, pathways, and cell types, offering significant advancements over traditional methods.

We applied SC-VAR to various diseases and traits using 12 scATAC-seq datasets. Our findings emphasize the critical role of epigenomic annotation in interpreting the regulatory functions of variants within noncoding regions. SC-VAR enabled us to uncover associations between genetic variants and genes across different tissues and developmental stages. For example, we found that SCZ risk genes are significantly enriched in neurodevelopmental processes in fetal brain cells, supporting the neurodevelopmental hypothesis of psychiatric disorders. This insight has potential implications for therapeutic strategies, particularly by highlighting the importance of early intervention. Additionally, SC-VAR identified disease-associated cell types and corresponding genes, which may guide targeted *in vitro* experiments to explore the relevant cellular mechanisms.

Our results have demonstrated SC-VAR’s effectiveness in identifying risk genes and cell types. SC-VAR outperformances traditional MAGMA in identifying more verified disease-related risk genes while having the similar type 1 error rates. The main advance of SC-VAR compared to MAGMA is that SC-VAR could identify more reliable regulatory element-related SNPs which are distal from the gene bodies. It is of note that H-MAGMA, which depends on three-dimensional chromatin interaction data (HiC) [15], is also designed to leverage enhancer-promoter links to interpret association of SNPs in regulatory region with disease. However, the HiC data are often not available for tissue samples and could not dissect cell types in heterogeneous tissue samples, while single-cell epigenomic data, especially scATAC-seq data, is currently available for many human tissues and organs, or can be much more easily obtained for most laboratories with low-cost commercial supports. Therefore, SC-VAR could be widely used to interpret genetic variants in clinical studies where single-cell epigenomic data can be easily obtained.

While SC-VAR offers a comprehensive framework for analyzing single-cell genomic data, it also faces challenges, particularly regarding computational demands and the need for tissue datasets that are not always available. In SC-VAR, the computation of cell-type associations is highly tissue-specific, indicating that the identified risk CREs are not readily transferable to data from similar tissues or sites. This tissue specificity underscores the importance of context when interpreting risk genes and regulatory elements, as the functional relevance of these associations may vary significantly across different biological environments. Additionally, the interpretation of complex multi-omics data requires expertise, which could limit its accessibility for some researchers. Although computational methods like SC-VAR offer valuable insights into disease-associated cell types and regulatory elements, they have inherent limitations in fully uncovering the intricate pathophysiology and mechanisms of diseases. Complementary biological experiments are crucial to validate these findings and provide deeper understanding. In the future, SC-VAR could be expanded to include more diverse types of single-cell data and further refined to increase its computational efficiency.

**Key Points**

- SC-VAR is a novel computational tool for predicting stage-specific, disease-associated risk genes, pathways, and cell types, using genome-wide association studies data and single-cell epigenomic data.
- SC-VAR outperforms state-of-the-art methods in terms of annotating more noncoding single-nucleotide polymorphisms (SNPs) located within distal *cis*-regulatory elements and predicting more risk genes and meaningful biological pathway.
- We have benchmarked SC-VAR on multiple complex diseases, demonstrating its capability in interpreting polygenic disease risks in a broad range of diseases.

## Supplementary Material

FigS1_bbaf123

Supplementary_Table_1_bbaf123

Supplementary_Table_2_bbaf123

Supplementary_Table_3_bbaf123

## Data Availability

SC-VAR software and source code are under MIT license and is freely available at Pypi (https://pypi.org/project/sc-var/). All codes used in this study and the manual are also provided in the GitHub repository (https://github.com/gefeiZ/sc_var/). Any additional information required to reanalyze the data reported in this paper is available from the lead contact upon request.
