# Supplementary figures and images for: SC-VAR: a computational tool for interpreting polygenic disease risks using single-cell epigenomic data
Source: Brief Bioinform. 2025 Mar 24;26(2):bbaf123. doi: 10.1093/bib/bbaf123 (PMC11932087; doi:10.1093/bib/bbaf123)

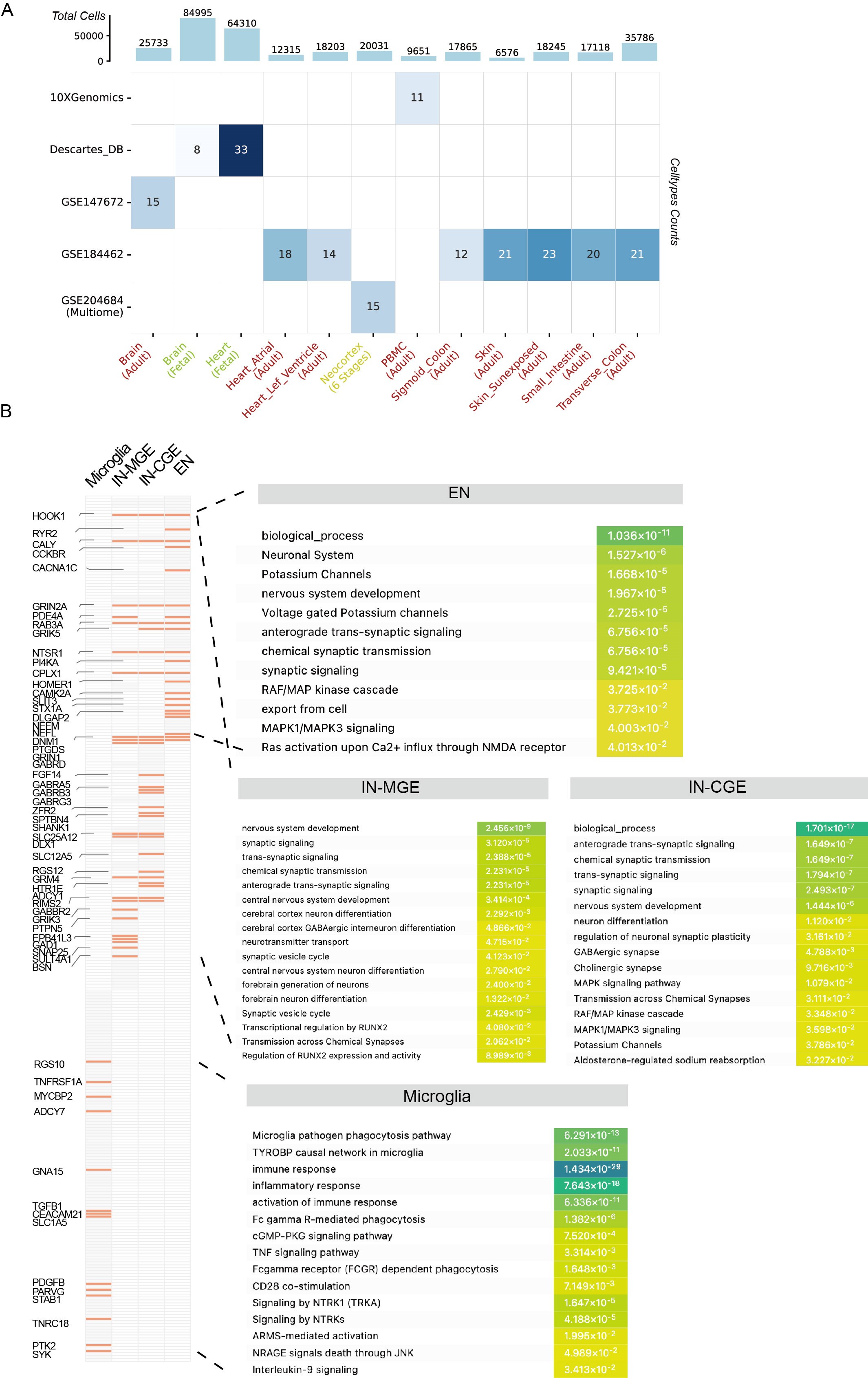

Supplement: FigS1_bbaf123 [file figs1_bbaf123.jpeg]
